# Cell Adhesion Molecules and Ubiquitination—Functions and Significance

**DOI:** 10.3390/biology5010001

**Published:** 2015-12-23

**Authors:** Mirka Homrich, Ingo Gotthard, Hilke Wobst, Simone Diestel

**Affiliations:** Department of Human Metabolomics, Institute of Nutrition and Food Sciences, University of Bonn, Katzenburgweg 9a, Bonn 53115, Germany; mhomrich@uni-bonn.de (M.H.); s7ingott@uni-bonn.de (I.G.); h.wobst@uni-bonn.de (H.W.)

**Keywords:** immunoglobulin superfamily, cell adhesion molecules, posttranslational modification, ubiquitination, endocytosis, intracellular trafficking

## Abstract

Cell adhesion molecules of the immunoglobulin (Ig) superfamily represent the biggest group of cell adhesion molecules. They have been analyzed since approximately 40 years ago and most of them have been shown to play a role in tumor progression and in the nervous system. All members of the Ig superfamily are intensively posttranslationally modified. However, many aspects of their cellular functions are not yet known. Since a few years ago it is known that some of the Ig superfamily members are modified by ubiquitin. Ubiquitination has classically been described as a proteasomal degradation signal but during the last years it became obvious that it can regulate many other processes including internalization of cell surface molecules and lysosomal sorting. The purpose of this review is to summarize the current knowledge about the ubiquitination of cell adhesion molecules of the Ig superfamily and to discuss its potential physiological roles in tumorigenesis and in the nervous system.

## 1. Introduction

Cell adhesion molecules are membrane-associated cell surface glycoproteins playing important roles in cell recognition, adhesion, migration and differentiation [1,2]. They can be subdivided into four different groups, which are defined by different structures and functional characteristics: the cadherins, integrins, selectins and immunoglobulin (Ig)-like proteins [3,4,5,6].

In this review we will focus on members of the Ig superfamily. They mediate calcium-independent cell adhesion and represent the biggest and structural most versatile group of cell adhesion molecules. One common component of all Ig superfamily members is the existence of at least one Ig-like domain in their extracellular region.

**Figure 1:**
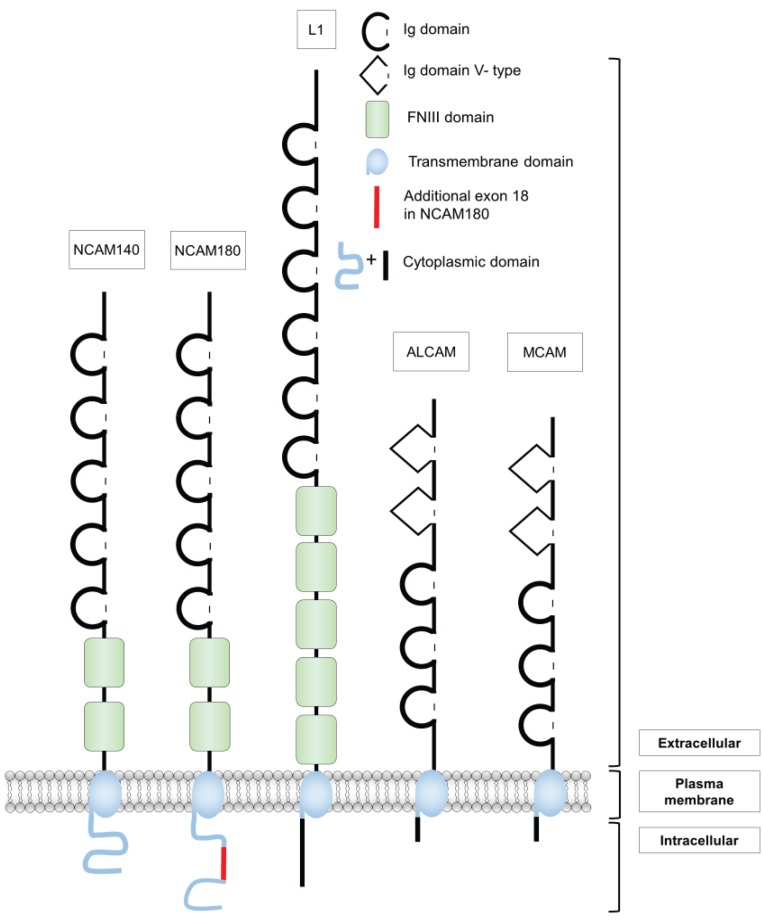
Overview about Immunoglobulin (Ig) superfamily members that are modified by ubiquitin. Ig superfamily members contain in their amino terminal extracellular domain Ig-like domains. Additionally, some of them have fibronectin (FN) type III domains (NCAM and L1). The ubiquitinated Ig superfamily members contain a transmembrane region and a cytoplasmic domain. NCAM: neural cell adhesion molecule; ALCAM: activated leukocyte cell adhesion molecule; MCAM: melanoma cell adhesion molecule; V: variable.

Usually, the members of this family contain big extracellular regions by which their adhesive function is mediated. Additionally, many of them contain a transmembrane region and a cytosolic domain which transmits signals into the cell thus providing intensive exchange of information [7]. Some Ig family members are attached to the plasma membrane via a glycosylphosphatidyl inositol (GPI) anchor. The extracellular fraction also contains secreted isoforms of Ig superfamily cell adhesion molecules that can be generated either by alternative splicing or cleavage by extracellular proteases. Some family members have additionally fibronectin (FN) type III domains in their extracellular region. In this review, we will describe in more detail the cell adhesion molecules NCAM, L1, MCAM and ALCAM since they play a role in the nervous system and in cancer and have been described to be ubiquitinated and discuss possible roles of this posttranslational modification (Figure 1). SHP substrate-1 (SHPS-1), another member of the Ig superfamily, was first shown to become ubiquitinated, however, since it additionally belongs to the signal-regulatory protein (SIRP) family, it will not be discussed in this context [8].

## 2. Structure and Functions of Different Cell Adhesion Molecules of the Ig Superfamily

### 2.1. The Neural Cell Adhesion Molecule NCAM

#### 2.1.1. Expression and Functions

The neural cell adhesion molecule NCAM was the first Ig superfamily member to be identified [9,10]. NCAM is widely expressed in the central and peripheral nervous system and in many other tissues but its function has mainly been investigated in the nervous system. Here, it is involved in processes like cell migration, synaptic plasticity, axonal growth and fasciculation [11,12,13,14,15,16,17,18,19]. A number of studies support a role of NCAM in the development of schizophrenia [20,21,22,23,24,25,26,27].

Several isoforms of NCAM are generated by alternative splicing with the three major isoforms being NCAM120, NCAM140 and NCAM180. NCAM120 is a GPI-anchored isoform, whereas NCAM140 and NCAM180 are transmembrane proteins. They are identical in their extracellular domains containing five Ig-like domains and two FN type III domains. NCAM140 and NCAM180 differ only in an alternatively spliced exon in their cytoplasmic tails leading to additional 261 amino acids in the NCAM180 isoform [28].

In the nervous system, the different isoforms exhibit different expression patterns with NCAM140 being mainly expressed on migratory growth cones and axon shafts of developing neurons but also on glia cells, whereas NCAM180 is enriched at sites of cell-cell contact and in particular at postsynaptic densities of mature neurons. In contrast, NCAM120 is preferentially expressed on glia cells [29,30,31].

In addition to the nervous system, NCAM is expressed in many other tissues and cell types like skeletal muscle cells, heart muscle cells, pancreatic endocrine cells, adult intestine, on natural killer cells and on peripheral blood T lymphocytes. It has also been reported to be expressed in lung epithelium in chicken [32,33,34,35,36,37,38,39].

NCAM also plays an important role in tumor progression. In some cancer types NCAM exerts a tumor-suppressive role (e.g., glial tumors), whereas, e.g. neuroblastoma and tumors of neuroendocrine origin, highly express NCAM, pointing to a tumorigenic effect [40,41,42,43,44,45]. Furthermore, abnormal NCAM expression has been shown in myeloma, gastrointestinal, thyroid, small lung cancer, epithelial ovarian and renal cancer [46,47,48]. Interestingly, in some tumors, NCAM switches from the NCAM120 isoform to NCAM140 or NCAM180 isoforms during tumor development [49,50,51,52,53]. The polysialylation (PSA), an extracellular posttranslational modification of NCAM (see below) also seems to play a role in tumorigenesis although its role seems to differ in different cancer types [42,54,55].

#### 2.1.2. Cellular Mechanisms

NCAM can interact with several molecules through its extracellular domain and its cytoplasmic tail [28]. Extracellular homophilic NCAM interactions can occur between NCAM molecules present on the same (*cis*-interaction) or on opposing cell surfaces (*trans*-interaction). On the cell surface, NCAM is present in a *cis*-dimeric form which is mediated by the first two Ig-like domains. These *cis*-dimers, in turn, mediate *trans* interactions. This *trans* interaction of NCAM *cis*-dimers is probably mediated by an interaction either between the second and third Ig-like domains or between all three N-terminal Ig-like domains [56]. Homophilic NCAM-NCAM interactions are important for cell adhesion, axonal fasciculation and NCAM-dependent neurite growth [57].

NCAM binds to several other molecules in a heterophilic mode including the Ig family members axonin-1/TAG-1 and L1. Binding to L1 induces phosphorylation of tyrosine and serine residues in L1 and stimulates neurite outgrowth [58,59,60]. Another important interaction partner is the fibroblast growth factor receptor (FGFR) whose direct binding to both FN modules of NCAM can activate FGFR signaling cascades thereby influencing neurite growth or tumor progression [61,62]. Additionally, it has been demonstrated that NCAM can act as a non-canonical ligand of FGFR1 and induce FGFR-dependent cell migration by promoting FGFR internalization with subsequent recycling of the FGFR to the cell surface [63,64]. The recently described interaction of NCAM with EphA3 regulates synapse formation [65,66]. NCAM also binds directly to glial derived neurotrophic factor (GDNF) and GDNF family receptor α-1 (GFRα-1), thereby functioning as an alternative signaling receptor for neurotrophic factors [67]. Further extracellular interaction partners of NCAM are components of the extracellular matrix like heparin, heparin-sulfate proteoglycans (HSPGs) and chondroitin-sulfate proteoglycans (CSPG) [68,69,70]. Interestingly, NCAM also binds prion protein (PrP) which recruits NCAM to lipid rafts and activates NCAM-dependent signal transduction via the non-receptor tyrosine kinase p59^fyn^ [71]. This interaction is required for neuronal differentiation of neural precursor cells of the subventricular zone (SVZ) [72]. A direct interaction with adenosine triphosphate (ATP) was also demonstrated [73].

Intracellular binding partners of NCAM include cytoskeletally associated proteins and signaling molecules. The first intracellular interaction partner to be identified was the cytoskeletal linker protein spectrin [74]. It binds to NCAM180 and NCAM140 and even indirectly to the GPI-linked NCAM120 isoform which is mainly confined to lipid rafts [75]. Spectrin builds a molecular bridge between NCAM and protein kinase C 2-β (PKCβ2). This indirect binding of PKCβ2 via spectrin to NCAM140 and NCAM180 also plays a role in NCAM-mediated neurite outgrowth [75,76,77]. Furthermore, GAP-43 (growth associated protein) could be co-precipitated with NCAM providing another connection to the cytoskeleton [78]. Further cytoskeletal interaction partners of NCAM140 and NCAM180 are α- and β-tubulin and α-actinin. The cytoplasmic proteins leucine-rich acidic nuclear protein (LANP), syndapin, the protein phosphatases PP1 and PP2A and phospholipase C-γ (PLC-γ) were also identified as interaction partners of NCAM140 and NCAM180 [79,80]. Specifically NCAM180 binds to microtubule associated protein 1A (MAP1A), β-actin, tropomyosin, RhoA-binding kinase-α and turned on after division-64 (TOAD-64). Recently, a direct interaction with the motor protein kinesin-1 could be demonstrated [79,80,81].

Since approximately twenty years it is known that NCAM can also act as a signaling receptor [82,83,84]. NCAM-dependent signaling can be initiated by homo- or heterophilic extracellular interactions (see above). Outside of lipid rafts NCAM activates the cAMP-dependent kinase (PKA) by yet unknown mechanisms [85]. Additionally, interactions with the FGFR activate PLC-γ leading to diacylglycerol (DAG) formation and subsequently to PKCβ2 activation, generation of arachidonic acid and elevated intracellular Ca^2+^ (calcium ions) [75]. In contrast, in lipid rafts NCAM binds constitutively receptor protein tyrosine phosphatase α (RPTPα) and the non-receptor tyrosine kinase p59^fyn^ [86,87]. Activation of p59^fyn^ by homophilic or heterophilic NCAM interactions leads to recruitment of focal adhesion kinase (FAK) to the p59^fyn^-NCAM complex and subsequently to activation of the ras-mitogen activated protein kinase (MAPK) pathway [77,88]. It seems that convergence of signaling from both NCAM fractions—raft and non-raft-associated—is required to allow cytoskeletal rearrangement and gene transcription cumulating in neurite outgrowth and cell migration [89]. All three major isoforms of NCAM are extensively posttranslationally modified. Both transmembrane isoforms NCAM140 and NCAM180 can be palmitoylated at cytosolic cysteine residues close to the transmembrane domain which is necessary for the correct distribution of NCAM in the plasma membrane and for correct intracellular signaling [89,90]. Different serine and threonine residues within the cytoplasmic domain can be phosphorylated [91,92]. Two so far identified kinases being responsible for NCAM phosphorylation are GSK-3 (glycogen synthase kinase-3) and casein kinase (CK) I [93]. NCAM contains one tyrosine residue in its cytoplasmic domain which can also be phosphorylated. This phosphorylation can be enhanced by direct association of NCAM with the receptor tyrosine kinase B (TrkB) and plays a role in NCAM-dependent neurite outgrowth [94,95]. However, NCAM is probably modified by several other, yet unknown kinases.

Additionally, the ubiquitin-fold modifier-conjugating enzyme-1 (Ufc1) interacts with the intracellular domain of NCAM140 [96]. Ufc1 is involved in the modification of proteins with the ubiquitin-like molecule ubiquitin-fold modifier-1 (Ufm1) and might therefore stimulate the ufmylation of NCAM. However, the exact mechanisms remain to be investigated.

A further important posttranslational modification is the extensive *N*-glycosylation in NCAM’s extracellular domain. Six potential glycosylation sites have been identified so far [97]. Most importantly NCAM is the major carrier for a unique modification, PSA which is linked *N*-glycosidically to two glycosylation sites in the Ig5 domain. In the brain, PSA is mainly expressed during development, reaches its maximum expression perinatally and is then drastically downregulated. In later developmental stages the PSA modification only remains in regions of the brain that maintain neurogenesis, including the SVZ, the granule cell layer of the hippocampus, particular regions of the hypothalamus and regions undergoing structural plasticity [98,99]. Several *in vitro* and *in vivo* studies suggest that PSA expression on NCAM converts NCAM from a molecule that promotes stability to one that promotes plasticity [100,101]. The PSA modification is also involved in NCAM’s effect on tumorigenesis but its role is discussed controversially. Depending on the tumor type, PSA seems either to reduce or to increase the tumorigenic potential [42,54,55].

Soluble NCAM forms are generated by different members of the disintegrin and metalloprotease (ADAM) family cleaving close to the plasma membrane resulting in an approximately 115 kDa fragment [18,19,102,103]. Shedding can be induced by tyrosine kinase and MAP kinase activity and has been implicated in neurite branching, outgrowth and cell migration [18,19,102]. Depending on the cell type, NCAM shedding either reduces or increases neurite outgrowth [19,102]. After induction of NCAM internalization another short extracellular 55 kDa fragment without any known function was observed, probably generated by a serin protease [104].

### 2.2. The Cell Adhesion Molecule L1

#### 2.2.1. Expression and Functions

Since its discovery in 1984 L1 has been established as a key player throughout the development of the nervous system [105]. In the developing nervous system it is widely expressed on postmitotic neurons, on astrocytes and on Schwann cells, in the adulthood on neurons and on cells of other tissues. L1 consists of six Ig-like domains, five FN type III domains, one transmembrane domain and a cytoplasmic tail and has a molecular mass of approximately 200 kDa. The molecular weight varies in different cell types dependent on different and extensive glycosylation at 22 potential *N*-glycosylation sites in L1’s extracellular domain. Different isoforms of L1 are generated by alternative splicing in a cell-specific manner. The isoform containing exons 2 and 27 represents the neuronal form of L1. Presence of exon 2 results in enhanced homophilic binding whereas amino acids RSLE encoded by exon 27 together with a preceding tyrosine result in a tyrosine-dependent sorting signal (YRSL) leading to clathrin-dependent endocytosis of L1 by interaction with the μ2-subunit of the adaptor protein 2 (AP-2) complex [106,107,108]. The non-neuronal isoforms without exons 2 and 27 are expressed in cell types like Schwann cells, hematopoetic cells and epithelial cells [109,110].

In addition to its function in neural development, L1 is also important for synaptic plasticity and regeneration in adult brain [111,112,113,114]. The importance of L1 is underlined by the occurrence of severe neurological disorders resulting from mutations in the human L1 gene collectively referred to as L1 syndrome [115,116,117,118,119].

Outside of the nervous system L1 has a major function during cancer progression. It has first been described in ovarian and endometrial carcinoma where its expression correlates with a poor prognosis [120]. Many subsequent studies revealed L1 expression in several tumor tissues. The function of L1 in tumorigenesis is also based on its ability to increase cell growth, motility, invasion and chemoresistance [121].

#### 2.2.2. Cellular Mechanisms

L1 interacts—like NCAM—with several molecules through its extracellular and cytoplasmic domain. L1 is likely to be part of a complex that involves *cis* and *trans* interactions at the cell surface thereby modulating L1 binding or activity [122]. In the nervous system, homophilic *trans*-interactions are important for axonal fasciculation and have been shown to have neurite growth promoting effects [123].

Heterophilic extracellular interaction partners include integrins, axonin-1/TAG-1, neurocan and phosphocan, neuropilin-1, F3/F11/contactin, ALCAM/DM-GRASP, CD24, NCAM, laminin and the FGFR [58,124,125,126,127,128,129,130,131,132,133,134,135,136,137,138]. The functions of heterophilic interactions of L1 have not yet fully been clarified. They have been suggested to enhance effects of homophilic L1 interactions. In this context, axonin-1/TAG-1 seems to play a role in L1-dependent neurite growth [130,131]. The *cis*-interaction of L1 with NCAM enhances L1 *trans*-interaction and concomitantly L1-stimulated cell aggregation, cell migration and neurite growth [58,60,139,140].

Additionally, the cytoplasmic tail of L1 provides linkages to the cytoskeleton and is associated with several kinases. One connection to the cytoskeleton is mediated by two binding sites in L1 for ERM family members which include the proteins ezrin, radixin and moesin (ERM). These molecules are crosslinkers between the membrane and the cytoskeleton. The first binding site for ezrin encompasses the Y^1176^RSLE sequence which is also involved in binding of the AP-2 complex (see above) [141]. The sequence KGGKY^1151^ located close to the transmembrane domain represents the second binding site to ezrin and has earlier been described to act as a linker to actin [142,143]. The phosphorylation status of the respective tyrosine residue of L1 (Y^1151^ by a src family member or Y^1176^ by pp60^c-src^) seems to be relevant for the interaction with ezrin [143,144]. This interaction may decrease lateral movement of L1 in the plasma membrane and thus regulate L1-dependent neurite outgrowth and branching [141,143,144]. Phosphorylation of S^1152^ by p90^rsk^ is important for axon outgrowth mediated by L1 and might also be involved in the regulation of ezrin binding [144,145].

As mentioned above, phosphorylation of Y^1176^ tightly regulates AP-2 binding in addition to regulation of ezrin binding. In the phosphorylated form, AP-2 cannot bind to the consensus sequence YRSL whereas in the non-phosphorylated form a binding site for AP-2 is created and clathrin-dependent endocytosis is initiated [146,147]. Endocytosis and cell migration also seem to be influenced by phosphorylation at S^1181^ which represents a casein kinase II consensus motif [148]. After endocytosis the major part of L1 is recycled to the cell surface. The endocytosis and recycling take place in the migratory growth cone where it is implicated in neurite outgrowth and polarized adhesion [106,149,150,151]. Endosomal trafficking of L1 is also required to target L1 from the somatodendritic compartment to the growing axon by transcytosis [152]. Recently, Rabex-5, a multidomain protein that has guanine nucleotide exchange factor (GEF) activity for Rab5, has been shown to bind to L1 and to regulate L1 internalization [153,154].

L1 also binds reversibly to ankyrin, an actin/spectrin adapter protein through a highly conserved FIGQY^1229^ motif in the cytoplasmic domain of L1 [155,156,157,158]. This interaction promotes stationary behavior of cells in culture and neurite initiation by inhibiting retrograde actin flow [159,160]. Phosphorylation of Y^1229^ by a yet unknown kinase disrupts L1-ankyrin binding resulting in enhanced neurite outgrowth *in vitro* and altered neuronal branching which leads to a decrease in perisomatic synapses of inhibitory GABAergic interneurons during cortex development [160,161,162,163,164]. This conserved motif also mediates the binding of L1 to the microtubule-associated protein doublecortin in the phosphorylated form [165].

These data show that phosphorylation of L1 by several kinases regulates intracellular binding. As for several other cell adhesion molecules, the involvement of L1 in signaling pathways is extremely complex. L1 has been shown to be phosphorylated *in vitro* and *in vivo* at several sites and these interactions are essential for L1 function. L1 crosslinking at the cell surface activates the MAP kinase extracellular signal-regulated kinase 2 (ERK2) which in turn phosphorylates S^1204^ and S^1248^ and goes along with L1 endocytosis [146]. Sustained activation of ERK2 by L1 crosslinking leads to increased motility and invasion into the surrounding matrix [166]. ERK activation is mediated by pp60^c-src^, phosphoinositide 3 kinase (PI3K), the Vav2 guanine nucleotide exchange factor, Rac1 GTPase and p21 activated kinase (PAK1) [146,167]. A fragment of L1 becomes additionally posttranslationally modified by small ubiquitin-like modifier (SUMO), which is necessary for its nuclear import [168].

The extracellular interaction of L1 with the FGFR is implicated in activation of FGFR signaling pathways and leads to L1-dependent neurite outgrowth via activation of PLC-γ, release of arachidonic acid and subsequent opening of voltage-gated Ca^2+^ channels as also shown for NCAM [169,170,171,172,173]. Ran binding protein in the microtubule-organizing center (RanBPM) was also identified as an L1 interacting protein and seems to serve as an adaptor in L1-mediated signaling in neurite growth [174,175]. Another mechanism of L1 signaling depends on its extracellular interaction with neuropilin-1 and semaphorin 3A (Sema3A), which induce recruitment of FAK to L1 and subsequent ERK activation resulting in growth cone collapse [176]. Finally, CK II co-precipitates with L1 and phosphorylates L1 constitutively at S^1181^ *in vitro* [177]. Since S^1181^ is located directly behind the YRSL motif an implication in L1 intracellular trafficking has early been suggested and its implication in endocytosis shown later on [148,178].

More complexity to L1 function is added by its extracellular and intramembranous cleavage by different proteases releasing soluble L1 fragments into the extracellular space thereby modulating cell migration of tumor cell lines and neurite outgrowth of neurons. Constitutive and induced cleavage of L1 generate fragments of 200, 140, 135, 80, 70, 32 and 28 kDa molecular weight, respectively [134,168,179,180,181,182,183,184,185].

### 2.3. The Melanoma Cell Adhesion Molecule MCAM

#### 2.3.1. Expression and Functions

The melanoma cell adhesion molecule MCAM has first been described in 1987 by Lehmann *et al.*, as a cell surface marker specific on melanoma cells but not on benign melanocytes [186]. Later on, it was classified as a cell adhesion molecule with a postulated tumorigenic potential [187]. MCAM is broadly and highly expressed in embryonic tissue but more limited in adult tissue where it is present, e.g., in smooth muscle cells, hair follicular cells, activated T cells, intermediate trophoblasts, mesenchymal stem cells and dental pulp [186,188,189,190,191,192,193]. Its expression can be induced by environmental signals thus initiating appropriate MCAM-specific reactions [194]. Its overexpression in several carcinoma tissues, e.g., prostate carcinoma, choriocarcinoma, angiosarcoma, Kaposi’s sarcoma, and leiomyosarcoma suggested a potential role in tumorigenic processes [195,196].

MCAM consists of a short cytoplasmic domain, a single transmembrane domain, and an extracellular domain containing five Ig-like units (V-V-C2-C2-C2) [186,197]. In its extracellular domain it contains eight putative *N*-glycosylation sites [198]. Different isoforms of MCAM have been described, a long form and a short form with molecular weights between 113 and 119 kDa, and a soluble form [198,199,200,201]. The long and short forms are identical in their extracellular domains and the transmembrane region, but differ greatly in the amino acid composition in their cytoplasmic tail. These isoforms are generated by alternative splicing whereas the soluble form seems to be either generated by alternative splicing or membrane shedding by a metalloprotease [198,202,203].

MCAM has been functionally implicated in cell adhesion, cell migration, proliferation, differentiation, signaling and immune response [194]. Since MCAM is mainly expressed in developmental stages it has been suggested to play a role in developmental processes. Consistently, it has been described to support adherence between neurons and glia cells with endothelial cells since it is mainly expressed on blood vessels but not on neural cells. Thus, by increasing vascularization of the tissue it facilitates development of the cerebellum and the peripheral nervous system [204,205,206,207]. It is also involved in organogenesis and maintenance of organ function like in the kidney and thymus and has been shown to be important for retina development in quail [208,209,210,211,212,213,214].

Furthermore, it has been implicated in inflammatory processes since it seems to play a role in recruitment of activated T cells to inflammatory sites and is upregulated in various inflammatory diseases [194,200].

An early study reported that in human tissues more than 70% of melanoma metastases express MCAM and that its expression correlates with increasing tumor thickness [215]. Later studies found that MCAM is overexpressed in most malignant and metastatic cancer and plays a significant role in tumor progression [194,216,217,218]. The function of MCAM is based on its ability to modulate cell motility and adhesion. In this context, it has been shown to increase motility and invasiveness of many tumor cells *in vitro* and metastasis *in vivo* by altering several apoptotic proteins involved in cell survival, proliferation and angiogenesis. Specifically, it was recently demonstrated that MCAM is involved in reduction of caspase-activation probably via its interplay with ALCAM [219]. Additionally, it elevates levels of the vascular endothelial growth factor, vascular endothelial growth factor receptor 2 (VEGF, VEGFR2) and CD31 as alternative ways to affect tumor metastasis [220]. Another study reported that inhibiting MCAM expression in melanoma cells may lead to loss of gap-junction communication, as evidenced by reduced invasion in a three-dimensional skin reconstruct model [221]. Furthermore, an MCAM antibody decreases melanoma cell invasion, tumor vascularization, matrix metalloproteinase-2 (MMP-2) expression and reduces formation of lung metastasis in nude mice [222].

#### 2.3.2. Cellular Mechanisms

MCAM interacts heterophilically with some recently identified molecules like laminin-411, Galectin-1, Wnt5a, the VEGFR2 and the neurite outgrowth factor [223,224]. It also mediates homophilic interaction that is involved in neurite extension and neuron development probably by modulating cell–cell adhesion [201,224,225,226,227,228,229,230,231]. However, in contrast to other well analyzed cell adhesion molecules not much is known about interaction partners of MCAM.

In the cytoplasmic domain MCAM contains a conserved positively charged KKGK motif mediating interaction with proteins of the ERM family. This interaction leads to recruitment of Rho GDP-dissociation inhibitor (RhoGDI) 1, activation of RhoA and results in increased cell migration [232]. MCAM induces microvilli formation in lymphocytes and increases the rolling and adhesion of lymphocytes on endothelial cells. The microvilli formation depends on the interaction of the long MCAM isoform with the actin cytoskeleton and requires a PKC phosphorylation site in the cytoplasmic tail [200]. A double leucine motif in the cytoplasmic tail is responsible for basolateral targeting in epithelia [194].

Additionally, MCAM can—like other cell adhesion molecules—activate signal transduction cascades. A reciprocal relationship between MCAM expression and AKT phosphorylation in melanoma cells has been suggested. Blocking AKT activation results in decreased MCAM expression, while overexpression of MCAM, likewise, increases phosphorylation of AKT and of the pro-apoptotic protein Bad thus becoming inactive [233,234]. In addition, engagement of MCAM in endothelial cells leads to activation of p59^fyn^ and subsequent tyrosine phosphorylation and activation of FAK. Paxillin can also bind to activated FAK and thus increase cell motility [235]. Additionally, MCAM triggering initiates PLCγ-mediated Ca^2+^-influx resulting in activation of PYK2 and p130^CAS^ [236]. The p38 kinase can be activated as a response to MCAM triggering leading to activation of the NFκB cascade which is critical in cell migration, angiogenesis and tumor metastasis [237,238]. MCAM has also been suggested to stimulate the transcription factors c-fos and c-jun [239]. Overall, these results demonstrate that MCAM plays a role in both cell–cell interactions and signal transduction in tumor cells.

### 2.4. The Activated Leukocyte Cell Adhesion Molecule ALCAM

#### 2.4.1. Expression and Functions

Another member of the Ig superfamily is the cell adhesion molecule ALCAM/DM-GRASP, which was first described by Burns *et al.* [240]. The name DM-GRASP was originally derived from its detection by antibodies recognizing an immunoglobulin-like restricted axonal surface protein. The prefix DM originates from expression of DM-GRASP in the dorsal funiculus and ventral midline of the chick spinal cord. DM-GRASP has several synonyms including SC1, BEN, ALCAM and CD166 [241,242,243]. In this review, the term ALCAM will further be used for all homologues in all species. ALCAM is a glycoprotein with eight putative *N*-glycosylation sites [244]. The molecular weight of the mature form has been described between 95 and 110 kDa [240,244]. In its extracellular domain ALCAM contains five Ig-like domains (V-V-C2-C2-C2), a transmembrane region and a short cytoplasmic domain [243,245]. A soluble splice variant of ALCAM consisting of the single amino-terminal V-type l Ig-like domain which is required for cell–cell-adhesive interactions was detected in endothelial cells [246]. It can also be cleaved in its extracellular domain close to the plasma membrane by ADAM17 resulting in a secreted form which has been suggested to reduce cell–cell adhesion in cancer [247,248]. ALCAM has first been detected in chicken on a restricted population of axons and was later found on many other cell types like activated leucocytes, hematopoetic stem cells and myeloid progenitors [240,249]. It has also been detected on neuronal cells, mesenchymal stem cells and bone marrow stromal cells and is expressed in most developing tissues like the central and peripheral nervous system, sensory organs, during hematopoiesis and in endothelial and epithelial lineage pointing to a function of ALCAM in many developmental processes [245,249,250,251,252,253]. Indeed, loss of ALCAM results in loss of cell adhesion and in developmental defects [254]. Although it is additionally expressed in several multipotent cells including cells of umbilical cord blood, bone marrow, testes, fetal lung, intervertebral disc and dental pulp [255,256,257,258,259,260]. It is not yet known if ALCAM contributes functionally to the multipotent capacity of these cell types [254].

ALCAM is also expressed in almost all cancers. *In vivo* mouse studies suggest a role of ALCAM in cancer progression [261,262,263]. However, depending on the original tissue of the cancer cells ALCAM becomes either upregulated or downregulated compared to healthy tissue, complicating the understanding of its role in cancer [264]. Due to its expression in cancer cells it is used more and more frequently as a biomarker of cancer progression in several tumor types like prostate cancer, colorectal cancer, breast cancer, oral cancers, pancreatice cancer, neuroblastoma, ovarian cancer and melanoma [265,266,267,268,269,270,271,272,273,274,275].

Several studies investigated ALCAM’s role in the nervous system. In neurons it plays a role in cell adhesion, axonal growth and navigation, in neuronal cell migration, differentiation and synapse formation and specifically serves as a guidance molecule for cellular migration and neuronal outgrowth during development. Consistently, ALCAM-deficient mice exhibit delayed maturation of neuromuscular junctions and defects in axon fasciculation [240,250,276,277,278,279,280,281,282,283,284,285]. Interestingly, ALCAM is expressed on growing retinal ganglion cell (RGC) axons and provides a permissive signal for axon guidance of RGCs [249,278,279]. ALCAM’s functional significance in RGCs is highlighted by the fact that it is translated in growth cones which depends on ERK and target of rapamycin (TOR) activation and that this local translation is crucial for the preference of RGC axons on ALCAM substrate [286]. Recently, ALCAM has also been shown to function in axonal growth of dorsal root ganglion neurons and to modulate neurotrophin signaling thereby regulating neurotrophin-dependent neurite outgrowth [287,288]. ALCAM has also been associated with multiple sclerosis susceptibility although the mechanism is controversially discussed [289,290].

#### 2.4.2. Cellular Mechanisms

In addition to homophilic interactions via the amino-terminal V-type Ig-like domain ALCAM is able to mediate heterophilic bindings with L1/NgCAM and CD6 [129,276,291,292]. The heterophilic binding to CD6 is more robust and persistent than the homophilic ALCAM interaction and plays a role in activation of T cells and transmigration of leukocytes across the blood brain barrier. Another important prerequisite for this mechanism seems to be its concentration in cholesterol-enriched membrane microdomains of leukocytes [293,294,295,296,297,298,299]. In neurons the heterophilic interaction with L1 seems to be more important than other extracellular interactions and has been suggested to play a role in guidance of retinal axons during development [129,279,285]. Both, the heterophilic and homophilic ALCAM interactions are mediated by the *N*-terminal Ig-like domains of ALCAM (*trans* interactions) whereas lateral oligomerization of ALCAM at the cell surface (*cis* oligomerization) is mediated by the three membrane proximal C-type Ig-like domains [291,300]. Together, *cis*-oligomerization at the cell surface and *trans* interactions between adjacent cells would synergistically promote ALCAM recruitment and network formation at sites of cell-cell contact [253].

Homo- and heterophilic binding of ALCAM and hence ALCAM-dependent cell adhesion depends on PKCα [301]. In this context it is interesting that the short cytoplasmic domain of ALCAM does neither contain any known adaptor-binding sites nor consensus sequences for potential interaction partners. Nevertheless, a potential interaction and functional involvement of ALCAM and α-catenin have also been suggested [302].

The Rac-specific guanine nucleotide exchange factor Tiam1 which is one important regulator of Rho GTPase functions in tumor cells is also functionally involved in ALCAM-dependent cell adhesion and reduces cell migration in metastatic melanoma cells [303,304]. ALCAM can—like other members of the Ig superfamily—be internalized via a clathrin-dependent mechanism after ligand binding in the extracellular region followed by its recycling to the cell surface. This process has been discussed as a potential therapeutic mechanism to deliver immunotoxins into tumor cells [305].

Table 1 and Table 2 give an overview about the mentioned posttranslational modifications and interaction partners of the described cell adhesion molecules.

**Table 1:** Overview about the above mentioned posttranslational modifications of the described cell adhesion molecules. EC, extracellular; IC, intracellular; * form of *N*-glycosylation.

Cell Adhesion Molecule	Modification	References
**NCAM**	Polysialylation * (EC)	[306,307]
	*N*-glycosylation (EC)	[97]
	Palmitoylation (IC)	[90]
	Phosphorylation (IC)	[91,92,93,94,95]
	Ufmylation (IC)	[96]
**L1**	*N*-glycosylation (EC)	[308,309,310,311]
	Phosphorylation (IC)	[144,145,146,147,148,161,162,163,308,312]
	Sumoylation (IC)	[168]
**MCAM**	*N*-glycosylation (EC)	[198]
	Phosphorylation (IC)	[200]
**ALCAM**	*N*-glycosylation (EC)	[243,244]

**Table 2:** Overview about the above mentioned interaction partners of the described cell adhesion molecules. EC, extracellular; IC intracellular.

Cell Adhesion Molecule	Interaction Partners	References
**NCAM**	β-actin (IC)	[80]
	α-actinin (IC)	[80]
	ADAM (EC)	[19,102,103]
	ATP (EC)	[73]
	Axonin-1/TAG-1 (EC)	[59]
	CKI (IC)	[93]
	EphA3 (EC)	[65,66]
	FGFR (EC)	[61,63,64]
	GAP-43 (IC)	[78]
	GFR-α1, GDNF (EC)	[67]
	GSK-3 (IC)	[93]
	Heparin, HSPGs, CSPGs (EC)	[68,69,70]
	Kinesin-1 (IC)	[81]
	L1 (EC)	[58]
	LANP (IC)	[79]
	NCAM (EC)	[313]
	MAP1A (IC)	[80]
	p59^fyn^ (IC)	[86]
	PLC-γ (IC)	[79]
	PP1, PP2A (IC)	[79]
	PrP (EC)	[71,72]
	RhoA-binding kinase-α (IC)	[80]
	RPTPα (IC)	[87]
	Spectrin (IC)	[74]
	ST8SiaII, ST8SiaIV	[314]
	Syndapin (IC)	[79]
	Tropomyosin (IC)	[80]
	TOAD-64 (IC)	[79]
	TRKB (IC)	[95]
	α- and β-tubulin (IC)	[80]
	Ufc-1 (IC)	[96]
**L1**	ADAM (EC)	[134,179,181,183]
	AP-2 (μ-subunit) (IC)	[178]
	ALCAM/DM-GRASP (EC)	[129]
	Ankyrin (IC)	[156]
	Axonin-1/TAG-1 (EC)	[130,131]
	CD24 (EC)	[128]
	CK II (IC)	[177]
	Doublecortin	[165]
	Erk2	[146]
	ERM proteins (IC)	[141,142]
	F3/F11/contactin (EC)	[124]
	FGFR (EC)	[138]
	Integrins (EC)	[315]
	L1 (EC)	[123]
	Laminin (EC)	[125]
	NCAM (EC)	[58]
	Neurocan (EC)	[126]
	Neuropilin-1 (EC)	[136]
	P^90rsk^ (IC)	[145]
	Phosphocan (EC)	[127]
	Rabex-5 (IC)	[154]
	(RanBPM)	[175]
**MCAM**	Actin (IC)	[200]
	ERM family	[232]
	Galectin-1 (EC)	[316]
	Laminin-411 (EC)	[317]
	MCAM (EC)	[224]
	Neurite outgrowth factor (EC)	[318]
	VEGFR2 (EC)	[319]
	Wnt5a (EC)	[320]
**ALCAM**	ADAM17 (EC)	[247]
	ALCAM (EC)	[276,321]
	CD6 (EC)	[292]
	L1 (EC)	[129]

## 3. Structure and Function of Ubiquitin

Ubiquitin is a polypeptide consisting of 76 amino acids. It exists in all eukaryotic cells and is highly conserved from yeast to humans. It is covalently attached to the ε-amino group of lysine (K) residues of target proteins. Alternatively, it can also be attached to the amino-terminal region of the target protein [322,323,324]. The attachment of ubiquitin is an ATP-dependent, three-step process: in the first step ubiquitin is activated by forming a thioester bond with a ubiquitin-activating enzyme (E1). Next, activated ubiquitin is transferred to a ubiquitin-conjugating enzyme (E2). Finally, ubiquitin-ligases (E3 enzymes) catalyze the transfer of ubiquitin with its carboxy-terminal glycine to a lysine residue of the target protein [325,326]. The E3 enzymes are one of the most important components in the ubiquitination process since they interact directly with the substrate and mediate in large part the ubiquitination specificity [327]. They can be classified into four different groups: the HECT (Homologous to the E6-AP Carboxyl Terminus) type, the RING (Really Interesting New Gene) type, the U-box type and the PHD (plant homeodomain)-finger type ligases. Thus far, 500–1000 different ubiquitin ligases are known [328,329]. Some ligases contain a phosphotyrosine-binding domain therefore only transferring ubiquitin to the responsible target protein after its tyrosine phosphorylation. This has intensively been studied for receptor tyrosine kinases which can be ubiquitinated by one of the Casitas b-lineage lymphoma (cbl) RING-type ubiquitin ligases [330]. Alternatively, phosphorylation activates binding of WW domain-containing ligases to target proteins as e.g. shown for CXCR-4 that binds AIP4/Itch ligase after phosphorylation [331]. Other E3 ligases do not bind directly to the target protein but rather need adapter proteins like members of the arrestin family that bind directly the cell surface protein. This has been shown, e.g., for neural precursor cell expressed, developmentally down-regulated 4 (Nedd4) ligase family members [332]. However, adding more complexity to this system, some E2 enzymes can transfer ubiquitin without interaction with an E3 ligase [333].

Ubiquitin contains seven lysine residues to which other ubiquitin molecules can be attached thereby generating ubiquitin chains of different length, structure and exhibiting different functions [334]. Therefore different types of ubiquitination are possible: mono-ubiquitination when only a single ubiquitin molecule is attached to a target protein; multiple mono-ubiquitination when ubiquitin is attached to several lysine residues of the target protein; and poly-ubiquitination when a ubiquitin chain is covalently linked to the target protein [335]. Classically, ubiquitin chains that are linked by K48 were considered as a signal for proteasomal degradation. Protein degradation by the proteasome is virtually involved in any cellular process. The proteasome is composed of a core particle (20S) containing multiple proteolytic sites and a regulatory 19S particle that is responsible for access of the substrates to the 20S core. After entrance into an internal chamber of the 20S particle ubiquitin is cleaved from the substrate to enter the cellular ubiquitin pool and the substrates are hydrolyzed [336]. The importance of proteasomal degradation is underlined by the development of several diseases attributable to genetic mutations in one of the components of the ubiquitin-proteasome system. Protein accumulation results in many diseases like several neurodegenerative disorders, cystic fibrosis, Liddle syndrome and many cancers [337]. Additional to its classical function, several studies discovered that K48-poly-ubiquitin chains also mediate non-proteolytical functions and further that all seven lysine residues of ubiquitin can be involved in building ubiquitin chains thus generating chains of different length and structure. It is now also known that the proteasome accepts other ubiquitin chains than K48-linked chains [338,339].

K63-linked ubiquitin chains are involved in many processes: protein trafficking by ubiquitination of cell surface proteins thereby directing them to the lysosomal degradation pathway [340]; DNA repair by ubiquitination of histone proteins resulting in non-homologues end joining and homologue recombination, respectively, after DNA damage [341,342]; inflammation e.g., by activation of the NFκB pathway by attachment of ubiquitin chains to different effectors within this cascade [343]; and regulation of ribosomal protein synthesis by attachment of ubiquitin to one of the ribosome subunits, L28 [344].

The functions of other ubiquitin-linked chains are not well understood. Some results suggested K11-linked ubiquitin chains preferentially in endoplasmic reticulum-associated degradation (ERAD), Tumor Necrosis Factor signaling and Anaphase-Promoting Complex-mediated proteolysis [345,346,347]. Additionally, K27- and K33-linked ubiquitin chains have been implicated in stress response and finally K29-linked chains seem to play a role in lysosomal degradation and ubiquitin fusion protein degradation [348,349,350]. Additionally, multiple mono-ubiquitination has been shown to be involved in protein trafficking whereas single mono-ubiquitination is needed for sorting membrane proteins to the endosome and lysosome [351,352,353,354].

Specifically, there is increasing evidence that ubiquitin can act either as an endocytosis or sorting signal for cell surface proteins. For its action as an endocytosis signal usually multiple ubiquitin molecules or K63-linked poly-ubiquitin chains are necessary. This is attributable to the weak affinity of proteins of the endocytic machinery to a single ubiquitin tag. Usually ubiquitin signals are recognized by proteins containing ubiquitin-binding domains (UBDs). Thus far, approximately 300 proteins belonging to 20 families have been identified and more and more UBD-domain-containing proteins are being discovered [354,355,356]. UBDs bind to hydrophobic patches of ubiquitin and—depending on the type of ubiquitination—the hydrophobic patches are more (K63-linked ubiquitin chains) or less (K48-linked ubiquitin chains) exposed [332]. Binding of adjacent ubiquitin molecules within a ubiquitin chain might increase the affinity to UBDs. Additionally, the cells use other supporting mechanisms for efficient binding of ubiquitinated cargo proteins [332]. The best characterized adapter molecules that link ubiquitinated proteins to the clathrin-dependent endocytosis are Eps15 and Epsin which prefer K63-linked chains although it has recently been shown that one ubiquitin molecule is sufficient to recruit Eps15 to endosomes [357,358,359,360,361]. Altogether, to act as a sorting signal multiple mono-ubiquitination is favored over mono-ubiquitination. Proteins that appear after their internalization in endosomes may contain ubiquitin chains which may function either as a better binding motif for sorting molecules or as a buffer against the function of de-ubiquitinating enzymes (DUBs) [362]. In this regard, it has also been hypothesized that ubiquitination of cell surface receptors and subsequent lysosomal degradation may also play an essential role in the prevention of recycling of non-functional proteins to the cell surface [363].

Furthermore, it has been observed that ubiquitin can be attached to substrate proteins at different steps of endocytosis. It may, for example, regulate the first endocytic steps if it is attached to proteins that are still present at the plasma membrane, whereas it can serve as a lysosomal degradation signal if attached after internalization [364].

Like other posttranslational modifications ubiquitination is a reversible process. While attachment of ubiquitin is mediated by E1, E2 and E3 enzymes the specific removal of ubiquitin is catalyzed by DUBs. Nearly 100 DUBs are encoded in the human genome and they belong to five different families [365]. In humans four members of the ubiquitin *C*-terminal hydrolases (UCH), 55 active members of ubiquitin-specific hydrolases (USP), four members of the Machado Joseph disease domain proteins (MJD), 14 members of the ovarian tumor proteins (OTU) and seven active members of the JAB1, MPN, MOV34 metalloenzymes (JAMM) have been identified [340]. One important function of DUBs is to prevent cell surface molecules from degradation by removing ubiquitin. After de-ubiquitination they will instead be recycled to the cell surface [366,367,368,369].

Altogether, these facts demonstrate that ubiquitination is a highly versatile and complex posttranslational modification whose function has not yet completely been understood. Much more research is necessary to clarify the details of the already known functions and biochemical characteristics of ubiquitin and to identify possibly further functions.

## 4. Ubiquitination of Ig Superfamily Members

### 4.1. Ubiquitination of NCAM

It was first described in 2007 that NCAM becomes ubiquitinated and that ubiquitination regulates NCAM’s endocytosis [370].

The first evidence for internalization of cell adhesion molecules was published by the group of Eric Kandel dealing with the NCAM homologue ApCAM in *Aplysia californica* [371]. They investigated mechanisms of long-term facilitation in *Aplysia* sensory neurons which can be modulated by repeated application of serotonin [372,373]. It has been shown that after treatment with serotonin the amount of ApCAM at the cell surface was decreased by approximately 50% and that the internalized ApCAM was sorted into prelysosomal-endosomal compartments, probably resulting in its lysosomal degradation. This result proved that a learning relevant neurotransmitter is able to stimulate receptor-mediated endocytosis of ApCAM. Concomitant with the downregulation of ApCAM, an increased expression of clathrin light chain was observed suggesting a possible clathrin-mediated endocytosis [374]. Clathrin-dependent but also lipid-raft-dependent endocytosis of NCAM140 and NCAM180 have been confirmed in later studies [370,375]. A constitutive internalization of ApCAM which like NCAM lacks a typical internalization sequence, was excluded [371,376]. After stimulation with serotonin ApCAM internalization appeared most prominently at sites of membrane apposition. The decrease of cell adhesion at these sites by removal of cell adhesion molecules is most likely important for defasciculation of the axonal processes potentially to favor synaptogenesis and therewith restructuring of the neuron architecture and memory formation [371,377,378].

In accordance with these early results, a reduced amount of NCAM180 was observed in the rat dentate gyrus 3–4 h after passive avoidance training which could be attributable to increased internalization and a possible subsequent degradation of NCAM as shown for ApCAM [371,376,379]. Minana *et al.*, demonstrated later on that NCAM140 and NCAM180 are inducibly internalized in astrocytes by a clathrin-dependent mechanism [375]. In 2007 the developmentally regulated internalization of NCAM140 and NCAM180 was shown in neuronal cells. Interestingly, NCAM140 endocytosis seems to be relevant in immature neurons, whereas endocytosis of NCAM180 seems to be important when axon-dendrite interactions become established indicating a regulatory role of NCAM in neurite outgrowth or synapse stability, respectively. However, in contrast to ApCAM most of the internalized NCAM is recycled to the plasma membrane and only a small amount is degraded in lysosomes [370]. This discrepancy may be explained by the different systems in which the internalization was shown or by the different stimulation methods of ApCAM’s/NCAM’s endocytosis. Furthermore, NCAM internalization was detected in the soma, neurites and growth cones suggesting a role of its endocytosis in the different compartments. It is conceivable that NCAM internalization in neurites and growth cones might facilitate neurite growth in brain development and/or enhance synaptic plasticity in adult brain by translocalization of NCAM to other cell surface sites.

**Figure 2:**
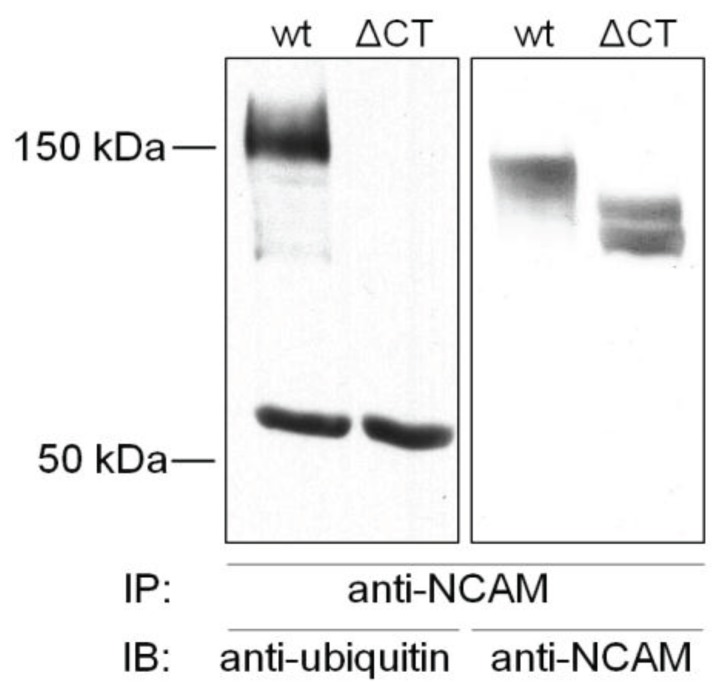
NCAM is ubiquitinated in the cytoplasmic region. B35 cells transfected with NCAM140 wild type (wt) or NCAM140 missing the entire cytoplasmic region (ΔCT), respectively, were lysed and subjected to immunoprecipitation (IP) with NCAM-specific antibodies. Immunoblot analysis (IB) was performed using ubiquitin-specific antibodies, recognizing mono- and polyubiquitinated proteins. The blot was reprobed with NCAM-specific antibodies as control.

Additionally, it was shown that NCAM is constitutively ubiquitinated with a drastic increase of ubiquitination after induction of NCAM endocytosis. Overexpression of ubiquitin resulted in increased endocytosis, whereas NCAM degradation was unaffected [370]. These results gave the first direct evidence that ubiquitination of NCAM acts as a signal for its endocytosis, although the amount of cell surface NCAM was not significantly reduced. This could be attributable to the intensive recycling of NCAM [370]. Therefore the function of NCAM internalization is probably not its downregulation but could rather result firstly in the removal of NCAM interacting molecules from the cell surface or secondly in its redistribution to other cell surface sites. Supporting the first hypothesis, PSA downregulation seems to depend on clathrin-mediated NCAM endocytosis in a rhabdomyosarcoma cell line [380]. Whether this is also true for neuronal cells needs further investigation. Otherwise, a translocalization to other cell surface sites has been demonstrated for L1, supporting the latter hypothesis [152]. In a previous study, it appeared that all three main NCAM isoforms, NCAM180, NCAM140 and the GPI-anchored NCAM120, could be modified by ubiquitin 3–4 h after passive avoidance training [379]. Ubiquitination occurs typically in the cytosol, although extracellular ubiquitination has also been described which could explain a ubiquitination of NCAM120 [381]. However, in neuronal cells a deletion construct of NCAM missing the entire cytoplasmic domain (ΔCT) is not ubiquitinated (Figure 2) excluding a possible extracellular ubiquitination of NCAM. Interestingly, in *Aplysia* only the membrane spanning isoform of ApCAM is internalized but not the GPI-anchored isoform [376]. Whether NCAM120 is internalized in neurons still needs to be investigated. However, ubiquitination of an interaction partner of NCAM120 which could be co-precipitated with NCAM could explain this result.

Analysis of the ubiquitination type of NCAM revealed an exclusive multiple mono-ubiquitination of NCAM at the plasma membrane [370]. In yeast, mono-ubiquitination was shown to be sufficient to target membrane proteins for internalization [382,383,384,385]. However, in more complex eukaryotes a single ubiquitin represents a very weak endocytosis signal [386,387]. For this reason, multiple mono-ubiquitination of NCAM fits perfectly into the described models. K63-linked ubiquitin chains can also act as endocytosis signals, however, since NCAM is not poly-ubiquitinated, this could not be the responsible mechanism for NCAM internalization [370]. 

Interestingly, ApCAM endocytosis is regulated by the phosphorylation of a MAP kinase consensus motif in its cytoplasmic tail. The same motif is present in NCAM and may also regulate NCAM’s endocytosis. Therefore, it has been speculated that phosphorylation of NCAM precedes its ubiquitination by creating a binding site for (a) ubiquitin ligase(s) leading to subsequent ubiquitination and endocytosis [370]. After its internalization the ubiquitin residues are most likely removed from NCAM in sorting endosomes by the DUB activity of the ubiquitin *C*-terminal hydrolase L1 (UCHL1) resulting in recycling of NCAM to the cell surface whereas a small portion of NCAM—which probably still contains covalently attached ubiquitin—is lysosomally degraded [388]. The action of UCHL1 has earlier been suggested to play a role in NCAM expression. The decrease of NCAM180 after passive avoidance training is accompanied by an increased expression of UCHL1, suggesting that ubiquitination is involved in memory consolidation. However, the results of this study proposed a ubiquitin ligase function of UCHL1 [379]. UCHL1 can act as a ubiquitin ligase in its dimeric form but interestingly, monomeric UCHL1 is well known as DUB that hydrolyses and detaches ubiquitin from proteins, which was confirmed for NCAM [388,389,390]. The different activities on NCAM in both studies may be explained by the dimerization state of UCHL1, the mono-ubiquitin stabilizing function of UCHL1 given by its ability to bind ubiquitin non-covalently and save NCAM from degradation or by the different approaches [391]. Interestingly, only the constitutive ubiquitination of NCAM was regulated by UCHL1 DUB activity, whereas the induced ubiquitination after activation of NCAM’s endocytosis was not altered. In line with this observation is that the endocytosis rate of NCAM remains unchanged after overexpression of UCHL1. Nevertheless, lysosomal degradation of NCAM was reduced after overexpression of UCHL1 supporting a role of UCHL1 in NCAM functions after its internalization. Since the triggering with NCAM antibodies leads to NCAM clustering at the cell surface and a possible conformational change in the cytoplasmic tail of NCAM, the accessibility of NCAM for UCHL1 could be reduced [88]. This would explain that only the constitutively ubiquitinated cell surface NCAM could be de-ubiquitinated by UCHL1. After internalization of NCAM an UCHL1 binding site might be exposed allowing its binding and therewith rescueing NCAM from lysosomal degradation. An observed partial colocalization of internalized NCAM and UCHL1 strengthens this hypothesis [388]. However, it cannot be excluded that other DUBs are involved in the regulation of NCAM’s intracellular trafficking. More research needs to be conducted to reveal other regulative factors for the regulation of NCAM ubiquitination and endocytosis and the physiological role of these processes in brain development and synaptic plasticity in adult brain.

### 4.2. Ubiquitination of L1

The ubiquitination of L1 was shown independently by different groups in primary neurons and different cell lines [154,392,393]. L1 is mainly mono- or multiple mono-ubiquitinated at the cell surface whereas only a small part seems to be poly-ubiquitinated [154,392]. The function of L1’s poly-ubiquitination is not yet known. Whereas the multiple mono-ubiquitination of NCAM is important for its endocytosis and does not play a role in its degradation, in L1 the mono- and/or multiple mono-ubiquitination seems rather to play a role in its lysosomal degradation as shown by co-localization and chemical inhibition studies [154,370,392]. The lysosomal degradation of L1 was mainly observed in the cell somata compared to neurites. Therefore, it has been speculated that the ubiquitination regulates the appearance of L1 in axons by either degrading it after attachment of ubiquitin or transporting it by transcytosis to the correct place [152,392]. Thus, ubiquitination of L1 might represent a fine regulation of L1-dependent growth cone motility and collapse [151,394]. In this context it would be interesting to identify responsible E3 ligase(s) and DUB(s) that are involved in reversible ubiquitination of L1 and to analyze the expression of these enzymes during growth cone motility and after synapse formation.

The effect of ubiquitination on L1 endocytosis seems to be less clear. Although it has been shown that overexpression of ubiquitin did not change the internalization rate of L1, it might be possible that the overexpression of ubiquitin cannot be effective since the other enzymatic components of the ubiquitin system (*i.e.*, E1, E2, and E3) are not overexpressed and may limit the entire process [370,392]. Otherwise, it has also been published that efficient internalization of L1 depends—at least partially—on its ubiquitination as demonstrated by using a ubiquitination-deficient L1 mutant [154]. However, the L1 mutant K11R missing all lysine residues used in this study may exhibit an altered three-dimensional structure of the cytoplasmic tail thereby changing other interactions necessary for optimal internalization. This aspect needs to be clarified in future studies.

Ubiquitination often depends on preceding phosphorylation thereby creating a binding motif for the respective E3 ligase(s) [395,396,397]. Consistent with this, phosphorylation of Y^1176^ in L1’s cytoplasmic tail plays a role in its ubiquitination. However, it is discussed controversially whether phosphorylation of Y^1176^ increases or decreases the ubiquitination of L1. Two different L1 mutants abolishing phosphorylation at position 1176 have been used to study the impact of Y^1176^ phosphorylation on ubiquitination level. The mutation Y^1176^ to phenylalanine exhibited reduced ubiquitination whereas mutation to alanine resulted in increased ubiquitination [154,392]. Both effects are conceivable: If phosphorylation precedes ubiquitination of L1 it is logical that a non-phosphorylated mutant exhibits lower ubiquitination levels than the wild type L1. This can only be clarified after identification of the responsible ubiquitin ligase. Another possibility is that AP-2 binding which is only possible in the non-phosphorylated form of Y^1176^ interferes with binding of the corresponding ubiquitin ligase thus explaining the reduced ubiquitination [147]. However, ubiquitination seems rather to interfere with ezrin-binding since ezrin does not co-precipitate with L1 after overexpression of ubiquitin [154]. A subsequent study showed that ubiquitination takes indeed place at the ERM binding site. Mutation analysis revealed that K^1147^ and/or K^1150^ of the cytosolic tail of L1 are the main ubiquitination sites of L1 [398]. It is furthermore imaginable that ubiquitin is attached as a result of endocytosis induction of L1 which would explain higher ubiquitination levels of L1Y^1176^A mutant. This would also explain the dependency between ubiquitination and the internalization rate as shown by Aikawa [154]. In this case, it is tempting to speculate that L1 ubiquitination might regulate lateral movement of L1 in the plasma membrane regulating neurite outgrowth and branching [141,142,143,144]. In agreement, it has been shown that Rabex-5 co-precipitates with L1, with higher affinity after induction of L1 endocytosis and with low affinity to a ubiquitin-deficient mutant. The interaction positively regulates L1’s internalization and seems to increase its ubiquitination thereby regulating endocytosis and proper postendocytic trafficking to lysosomes making the possible regulatory mechanisms more complex [154].

Altogether, subsequent studies are needed to investigate the cellular mechanisms of L1 ubiquitination and the role in L1-dependent functions in the nervous system and cancer development.

### 4.3. Ubiquitination of MCAM

Recently, it could be demonstrated that also MCAM can be ubiquitinated [219]. MCAM has classically been described in melanoma cells but it also seems to play a role in hepatocellular carcinoma via its interplay with ALCAM which can itself be ubiquitinated (see also Section 4.4) [399]. Earlier studies have shown that ALCAM is a valuable cancer stem cell marker in various cancer types and plays a role in tumor progression, e.g., in breast cancer [400,401,402,403]. ALCAM and MCAM in combination seem to be promising candidates as diagnostic markers for hepatocellular carcinoma and their crosstalk has intensively been investigated in a hepatocellular carcinoma cell culture model, the Bel-7402 cells [219,239,404]. In this cell culture model, ALCAM can regulate expression of MCAM at the level of degradation. It activates Akt which leads to subsequent phosphorylation and activation of c-Raf, MEK1 and ERK1 causing increased ubiquitination and hence downregulation of the two E3 ligases Smurf1 and βTrCP. These enzymes are responsible for ubiquitination of MCAM [219]. Therefore, by enhancing degradation of Smurf1 and βTrCP MCAM becomes less ubiquitinated and is stabilized at the cell surface explaining why downregulation of ALCAM decreases MCAM levels in Bel-7402 cells [219]. Interestingly, MCAM is the only cell adhesion molecule of the Ig superfamily for which ubiquitin ligases have been identified so far. In the same cell culture system expression of ALCAM negatively correlated with COP1, a RING type ubiquitin ligase. Therefore, COP1 could be a potential ubiquitin ligase regulating ALCAM degradation and hence its tumorigenic potential although further studies need to verify the involvement of COP1 in ALCAM degradation [405]. ALCAM is also ubiquitinated and subsequently downregulated in retinal ganglion cells from chicken (see also Section 4.4) indicating that ALCAM is degraded after ubiquitination independent from the species and cell culture system [399]. It has been shown for MCAM that it can be internalized after stimulation with its ligand Wnt5a resulting in its translocation to a polarized structure in a melanoma cell culture model. This process is necessary for cell orientation, polarity and directed migration [406]. It would be interesting to analyze whether MCAM endocytosis is regulated by ubiquitin. It has also not yet been investigated by which type of ubiquitination MCAM is modified or whether it is degraded lysosomally or by the proteasomal pathway. Nevertheless, the results showing a tightly regulated expression of MCAM by ALCAM and the ubiquitination of MCAM provides novel and highly interesting insights into the cellular regulation of MCAM and hence into hepatocellular carcinoma and potential future therapeutic approaches.

### 4.4. Ubiquitination of ALCAM

The ubiquitination of membrane-integrated ALCAM was first described in 2008 in retinal ganglion cells isolated from chicken [399]. In its cytoplasmic domain ALCAM contains seven lysine residues providing potential docking sites for ubiquitin. However, since ALCAM is probably mono- and/or di-ubiquitinated only one and/or two of the lysine residues seem to be used [399].

ALCAM is internalized via the clathrin-dependent pathway followed by recycling of ALCAM to the cell surface [305,399]. In cancer cells internalization of ALCAM seems to be necessary for rearrangement of cell–cell contacts since the maximum of internalized ALCAM has been observed at the cleavage furrow during cytokinesis [305]. In neurons, the endocytosis has been detected in the central domain of growth cones. Inhibition of clathrin-dependent endocytosis resulted in random axon growth in contrast to a control condition where axons grew preferentially on an ALCAM substrate proving that endocytosis of ALCAM is essential for axon navigation [399]. It has been hypothesized that also other members of the Ig superfamily use endocytosis to regulate their amount and localization at the cell surface in order to modulate neurite outgrowth and cell adhesion e.g., ApCAM, L1, and NCAM [149,150,152,370,376]. Therefore, it is conceivable that varying concentrations of ALCAM could represent one important mechanism in the development of the nervous system to regulate axonal outgrowth and pathfinding.

Interestingly, in cancer cells internalization of ALCAM happens much more slowly than in neurons. This fact supports a role of ALCAM internalization in rapid regulation of growth cone navigation in response to environmental changes. However, a small part of ALCAM in neurons seems to be internalized clathrin-independently and more slowly than the clathrin-dependent part of ALCAM. In leukocytes ALCAM is almost exclusively present in cholesterol-enriched microdomains and it is most likely that it exhibits the same distribution in other cell types [299]. However, it has been described that lipid rafts are excluded from the endocytic vesicles during clathrin-mediated endocytosis indicating that clathrin-mediated endocytosis takes place independent of lipid rafts [407]. On the other hand, it has been reported that clathrin-mediated endocytosis can be sensitive to cholesterol depletion indicating that the cellular mechanisms are not yet clear [408,409]. In this context, it would be interesting to investigate whether ALCAM undergoes clathrin-dependent or -independent endocytosis in the lipid-raft fraction.

Ubiquitination of ALCAM regulates the amount at the cell surface by enhancing its degradation probably by the lysosomal pathway as determined by analyzing the number and diameter of ALCAM-positive vesicles representing multivesicular bodies [399,410]. Overexpression of ubiquitin has no effect on the endocytosis of ALCAM although the authors discuss the possibility that an increased ubiquitination may not affect its internalization rate since it has been shown earlier that an enhanced ubiquitin pool does not affect internalization of (overexpressed) trans-membrane proteins. This could probably be due to sufficient endogenous ubiquitin levels already allowing optimal internalization. Mutation of corresponding lysine residues might solve this question.

Furthermore, it still needs to be investigated whether ubiquitination might affect the slow endocytosis of ALCAM which seems to be clathrin-independent [399]. Concomitantly, ubiquitin overexpression results in a higher percentage of cells with internalized ALCAM pointing to a potential role of ubiquitin in ALCAM’s slow endocytosis. Several reports support the view that ubiquitination might regulate either lipid-raft-dependent or clathrin-dependent endocytic pathways [360,386,410,411,412]. The authors discuss also that the kind of ubiquitination might influence the fate of ALCAM after its endocytosis: Di-ubiquitinated ALCAM would be more efficiently sorted and hence degraded than mono-ubiquitinated ALCAM since the affinity between ubiquitin-sorting receptor and ubiquitinated ALCAM is higher between two ubiquitin molecules and adapter proteins than one ubiquitin molecule and the respective adapter protein [413,414].

Altogether, more studies are needed to investigate the cellular mechanisms of ALCAM endocytosis and ubiquitination and the functional role of these processes. This aspect is also especially very interesting in cancer cells since ALCAM has been suggested to play a crucial role in cancer progression.

## 5. Conclusions and Perspectives

Some of the here described cell adhesion molecules have been known for a long time and have been analyzed in detail. However, their ubiquitination has only been shown in the last decade. One reason is most likely the ongoing ubiquitin research giving continuously more insight into the complexity and function of ubiquitin modifications.

**Figure 3:**
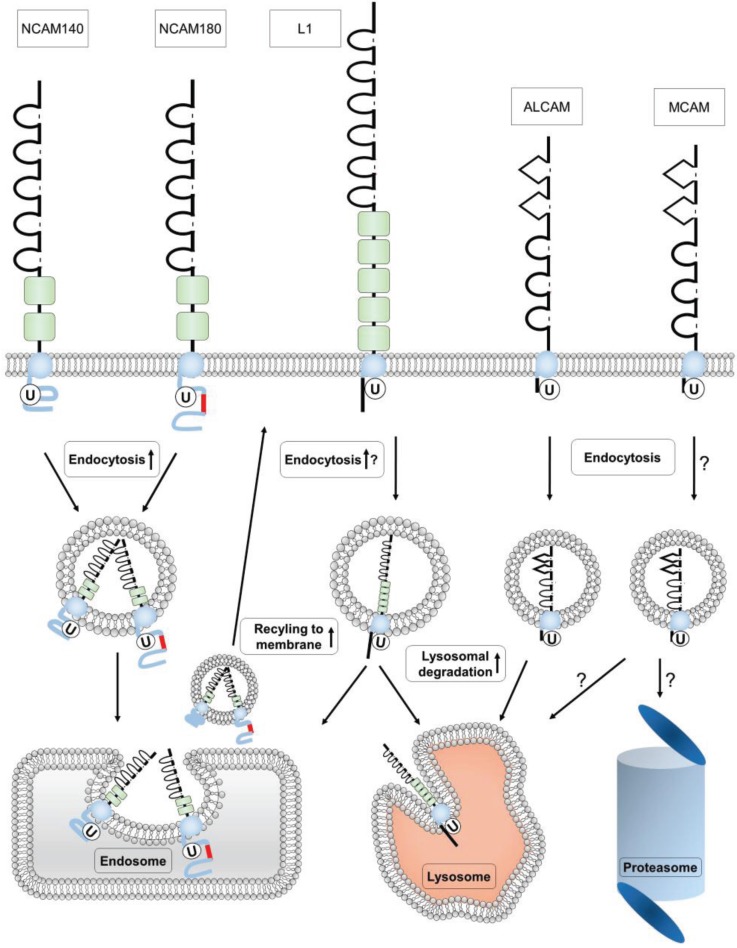
Overview about the function of ubiquitination for different Ig superfamily cell adhesion molecules. All depicted molecules are internalized after a specific stimulus. Ubiquitination (U) of NCAM increases its endocytosis (↑) but has no effect on its lysosomal degradation after overexpression of ubiquitin. However, cleavage of ubiquitin by the de-ubiquitinating enzyme UCHL1 results in decreased lysosomal degradation of NCAM thus favoring its recycling to the cell surface (↑). For L1 it could be shown that ubiquitination increases its lysosomal degradation (↑) whereas it is not yet clear whether it also upregulates its internalization (↑?). ALCAM’s endocytosis does not seem to be regulated by its ubiquitination although ubiquitin overexpression decreases ALCAM expression. This is most likely attributable to increased lysosomal degradation (↑). MCAM ubiquitination is mediated by the E3 ligases Smurf1 and βTrCP by a tight crosstalk with ALCAM-mediated signal transduction and leads to MCAM downregulation. The detailed mechanism of its degradation has not yet been investigated (?).

For the here described cell adhesion molecules of the Ig superfamily, ubiquitination plays a role in internalization and/or intracellular trafficking, *i.e.*, lysosomal degradation, transcytosis or recycling (Figure 3). The discovery that cell adhesion molecules can be posttranslationally modified by ubiquitin adds an additional regulatory factor for their functions. The here mentioned cell adhesion molecules play a role in the nervous system and in tumor development. In the nervous system, their ubiquitination might be implicated in the fine regulation of their presence at the cell surface and therefore allow rapid adaptation to environmental influences that occur during brain development or synaptic plasticity during learning processes. Therefore, it might be highly interesting to investigate other CAMs for their internalization and ubiquitination. In this context, SynCAM could be a favorable candidate for further analysis. It is expressed at synaptic connections and is critical for assembly, organization and maintenance of synapses [415,416,417]. Hence, it is tempting to speculate that a possible internalization and regulation by ubiquitin might regulate synapse development and stability.

In cancer cells, the ubiquitination might be responsible for the fine regulation of cell adhesion and migration of cancer cells thus being potentially involved in their metastatic potential. Further studies are needed to clarify these aspects. It would be further interesting to analyze whether different cellular processes are active in tumorigenic cells than in neural cells. An example of a different cellular mechanism in cancer is likely provided by the cell adhesion molecule “roundabout” (Robo). The Robo family represents, together with its ligands, the Slits, essential repellent axon guidance molecules during embryonic development and has several other functions [418,419,420]. Removal of Robo from the cell surface by clathrin-dependent endocytosis and subsequent lysosomal targeting results in inappropriate midline crossing [421]. Otherwise, proteasomal degradation of Robo after its ubiquitination regulates breast cancer cell migration indicating the involvement of a different cellular mechanism in cancer cells. Therefore, it would be interesting to analyze whether Robo ubiquitination also plays a role in embryonic neural development and which process is regulated by its possible ubiquitination [422]. Similarly, for the cell adhesion molecule “deleted in colon carcinoma” (DCC), a proteasomal degradation has been shown after ubiquitination; however, a role of ubiquitination in axonal pathfinding and/or cancer has not been investigated yet [423]. Even less is known for other cell adhesion molecules, like CHL1, an L1 related cell adhesion molecule. CHL1 undergoes endocytosis which is required for CHL1-dependent neuritogenesis. A possible regulation by ubiquitination has not been analyzed so far [424].

Altogether, although cell adhesion molecules have been analyzed since the 1970s the molecular mechanisms regulating the cellular functions of cell adhesion molecules are so far not well understood. Their ubiquitination represents a novel approach for understanding their function in more detail. Especially in tumorigenesis, their ubiquitination could represent a new avenue for potential therapies since the ubiquitination is highly specifically regulated by the ubiquitin ligases. In this context, some ubiquitin ligase inhibitors have already been developed, however, they target mainly proteins that are proteasomally degraded, e.g., p53, and there was not much clinical advance in treating selected tumors [327]. Furthermore, MCAM is the only Ig superfamily member for which ubiquitin ligases have been identified so far. Therefore, much more research is needed to identify the regulation of their ubiquitination (*i.e.*, identification of ubiquitin ligases (DUBs)) and to clarify the physiological role of ubiquitination of Ig family cell adhesion molecules in different cells and conditions.
